# Effect of different low-level intensity laser therapy (LLLT) irradiation protocols on the osseointegration of implants placed in grafted areas

**DOI:** 10.1590/1678-7757-2020-0647

**Published:** 2021-04-14

**Authors:** Guilherme José Pimentel Lopes de OLIVEIRA, Felipe Eduardo PINOTTI, Maurício Andres Tinajero ARONI, Elcio MARCANTONIO, Rosemary Adriana Chiérici MARCANTONIO

**Affiliations:** 1 Universidade Federal de Uberlândia Faculdade de Odontologia da Uberlândia Departamento de Periodontia/Implantodontia UberlândiaMG Brasil Universidade Federal de Uberlândia (FOUFU), Faculdade de Odontologia da Uberlândia, Departamento de Periodontia/Implantodontia, Uberlândia, MG , Brasil.; 2 Universidade Estadual Paulista Faculdade de Odontologia de Araraquara Departmento de Diagnóstico e Cirurgia AraraquaraSP Brasil Universidade Estadual Paulista (UNESP), Faculdade de Odontologia de Araraquara, Departmento de Diagnóstico e Cirurgia, Araraquara, SP, Brasil.; 3 Universidad San Francisco de Quito Departamento de Periodoncia Quito Ecuador Universidad San Francisco de Quito (UFSQ), Departamento de Periodoncia, Quito, Ecuador.

**Keywords:** Bone substitutes, Low-level intensity laser therapy, Osseointegration

## Abstract

**Objective:**

To evaluate the effect of different protocols of low-level intensity laser therapy (LLLT) irradiation on the osseointegration of implants placed in grafted areas.

**Methodology:**

84 rats were randomly allocated into six groups: DBB: defect filled with deproteinized bovine bone; HA/TCP: defect filled with biphasic ceramic of hydroxyapatite/β-tricalcium phosphate ; DBB-LI: defect filled with DBB and treated with LLLT after implant placement; HA/TCP-LI: defect filled with HA/TCP and treated with LLLT after implant placement; DBB-LIB: defect filled with DBB and treated with LLLT after graft procedure and implant placement; and HA/TCP-LIB: defect filled HA/TCP and treated with LLLT after graft procedure and implant placement. The bone defects were made in the tibia and they were grafted. After 60 days, the implants were placed. The rats were subsequently subjected to euthanasia 15 and 45 days after implant placement. The pattern of osseointegration and bone repair in the grafted area was evaluated by biomechanical, microtomographic, and histometric analyses. Furthermore, the expression of bone biomarker proteins was assessed.

**Results:**

The LLLT groups presented higher removal torque, mineralized tissue volume, and a greater degree of osseointegration, especially when LLLT was performed only after implant placement, and these findings were associated with higher expression of BMP2 and alkaline phosphatase.

**Conclusion:**

LLLT performed on implants placed in grafted areas enhances the osseointegration process.

## Introduction

The improvement in bone formation in grafted areas with osteoconductive bone substitutes may diminish the time for implant loading and positive long-term outcomes of the rehabilitation with implants. Although the use of osteoconductive bone substitutes reduces bone formation in bone defects,^1^ these bone substitutes have been used extensively, since the use of autogenous bone grafts is related to donor site morbidity.^2^

Low-level laser therapy (LLLT) has been successfully used in several clinical conditions, such as those involving joints,^3^ muscles,^4^ cutaneous tissue,^5^ and nerve tissue^6^ lesions. It has been proposed that the activation of mitochondrial chromophores stimulates the action of the respiratory chain with subsequent increase in cellular metabolism, producing the beneficial actions of LLLT in the process of tissue regeneration.^7^ The benefits of LLLT use have also been demonstrated in bone tissue by the stimulation of the differentiation and activation of osteoblastic cells.^8^ Preclinical studies have shown that the use of LLLT accelerated the repair of long bone fracture models,^9^ stimulated the healing of critical-sized calvarial defects,^10,11^ and accelerated the osseointegration of implants placed in native^12-15^ and grafted bone.^16-18^

Previous studies using LLLT in infrared wavelength range have shown improvement in the healing of grafted areas with different types of osteoconductive biomaterials.^11,19-21^ A preclinical study demonstrated that the use of LLLT at an 808 nm wavelength increased bone tissue formation in grafted areas with deproteinized bovine bone (DBB) and biphasic ceramics based on hydroxyapatite and β-tricalcium phosphate (HA/TCP) and that this effect was related to the increased expression of biological mediators that stimulate the formation of bone tissue.^22^

The use of LLLT aimed to accelerate osseointegration in grafted areas has not been previously explored. In an earlier study, our research group demonstrated that the use of LLLT to DBB and HA/TCP grafted areas in the tibia of rats improved the osseointegration process of implants.^18^ However, LLLT protocols to accelerate the osseointegration of implants in areas of native bone use LLLT sessions after implant placement.^13,14,23,24^ Moreover, the association of the use of LLLT sessions at two different times (after grafting and after implant placement) was also not described. Thus, this study compares the effect of different LLLT protocols on osseointegration in areas grafted with DBB and HA/TCP.

## Methodology

This study was submitted and approved by the Research Ethics Committee on Animal Use of our institution (08/2014) and it was conducted according to the international guiding principles for biomedical research involving animals and followed the ARRIVE guidelines. In total, 84 animals (*Rattus novergicus*, Hotzman strain) aged three months old and weighing 250-300 g were used. The animals were kept in an environment with controlled temperature (21±1°C), humidity (65-70%), and light-dark cycles (12 hours) and they had access to appropriate food and water *ad libitum.*

### Groups

**Figure 1:**
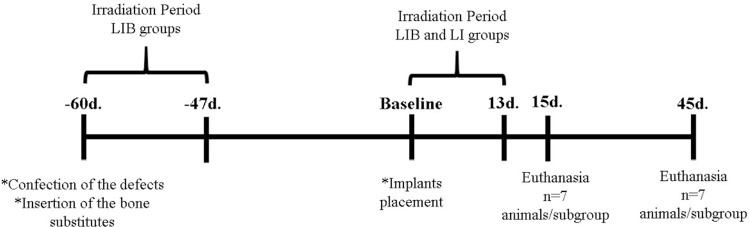
Flowchart of the experiment. LLLT irradiation began after implant placement in the LI groups and after grafting procedures and implant placement in the LIB groups. There were seven sessions that were repeated every 48 hours for 13 days. The implants were installed 60 days after the grafting surgery. The animals were euthanized after 15 and 45 days of implant placement

The animals were randomly distributed into six groups with 14 animals each according to the type of biomaterial and the LLLT protocol used: the DBB group: defect filled with deproteinized bovine bone graft (DBB) (Bio-Oss^®^, Geistlich AG, Wolhusen, Switzerland); the HA/TCP group: defect filled with biphasic ceramic based on hydroxyapatite/β-tricalcium phosphate (HA/TCP) (Straumann^®^ Bone Ceramic, Straumann AG, Basel, Switzerland); the DBB-LI group: defect filled with DBB and treated with LLLT after implant placement; the HA/TCP-LI group: defect filled with HA/TCP and treated with LLLT after implant placement; the DBB-LIB group: defect filled with DBB and treated with LLLT after the graft procedure and implant placement; the HA/TCP-LIB group: defect filled with HA/TCP and treated with LLLT after the graft procedure and implant placement. The bone defects and grafting procedures were performed 60 days before implant placement, and the animals were euthanized 15 and 45 days after implant placement. LLLT was performed after implant placement in the LI groups; in the LIB groups LLLT was performed after grafting procedures and implant placement. (Figure 1).

### LLLT protocol

A GaAlAs laser (Thera Lase, λ 808 nm, 100 mW, ϕ ∼0.60 mm, focal divergence 0.45 rad, CW, DMC Equipamentos, São Carlos, Brazil) was used to perform the irradiation. The grafted area was delimited after the sutures of the surgical site, aided by a tissue marker pen. Four equidistant 3-mm points were marked in order to encompass the whole area to be irradiated; these points also served as a guide for laser irradiation. The laser was irradiated with the laser tip in contact with the skin tissue for 10 seconds at each point (1 J), totaling 40 seconds of irradiation per session (4 J). Seven sessions were performed – which were repeated every 48 hours for two weeks after the grafting procedure or implant placement. The energy density at each point was approximately 354 J/cm^222^. The animals of the control groups were submitted to placebo LLLT interventions to handle the animals of every group with the same frequency.

### Surgical procedure

The surgical protocol was the same as that used in another preclinical study^18^. The animals were anesthetized by a combination of 0.08 ml/100 g body weight of ketamine (Agener União Ltda, São Paulo, Brazil) and 0.04 ml/100 g body weight of xylazine (Rompum, Bayer SA, São Paulo, Brazil). Subsequently, a trichotomy of the inner region of the right and left legs and disinfection with sterile gauze soaked in iodopovidone solution were performed.

A 10-mm incision was made in planes on the tuberosity of the tibial. After a delicate dissection, the bone tissue was subjected to osteotomy by a spherical drill mounted at a contra-angle with the aid of an electric motor adjusted to 1200 rpm under abundant irrigation of sterile saline solution. The defect formed had measurements of 4 mm in both length and width and 1.5 mm in depth. A periodontal probe was used to measure defects that were later filled with biomaterials. The tissue was sutured by planes internally with resorbable wire (5.0, Vicryl Ethicon, Johnson & Johnson, São José dos Campos, Brazil) and externally with silk thread (4.0, Ethicon, Johnson & Johnson, São José dos Campos, Brazil). The animals received a single dose of multibiotic at a dose of 0.3 ml/kg of body weight (Multibiotic Small, Vitalfarma, São Sebastião do Paraíso, Brazil) and ketoprofen at a dose of 0.3 ml/kg body weight (Ketoflex, Mundo Animal, Sao Paulo, Brazil).

After 60 days, a second surgical intervention was performed in the previously grafted area to place the implants. An incision similar to the first surgical procedure was performed on the tuberosity of the tibia. The grafted region was prepared for implant placement by applying a progressive sequence of milling drills (milling cutter, 2.0 mm spiral drill – Neodent^®^, Curitiba, Brazil) to accommodate a titanium implant with 4 mm high and 2.2 mm in diameter (Machined Surfaces, Neodent^®^, Curitiba, Brazil). All perforations were performed using an electric motor (BLM 600 - Driller, São Paulo, Brazil) adjusted to 1200 rpm under abundant irrigation of sterile saline solution. The implant was installed with the help of a digital key. The tissue suture and the postoperative drug protocol were similar to those used in the first surgery.

At 15 and 45 days after the surgical procedures for implant placement (Figure 1), the animals were subjected to euthanasia via an overdose of anesthetic. The tibiae were separated according to the analyses performed. The right tibia was used for microtomographic and histomorphometric analysis on the non-decalcified sections, whereas the left tibia was used for biomechanical analysis, histological description, and immunohistochemical analysis.

### Biomechanical analysis

After euthanasia, the left tibiae were stabilized in a small vice. A hexagonal wrench was attached to both the implant and the torque wrench (Tohnichi, model ATG24CN-S - with a graduated scale of 0.05 Ncm, measuring force from 3 to 24 Ncm), and an anti-clockwise movement was performed to unscrew the implant. The maximum peak needed to move the implant was noted as the removal torque value.

### Descriptive histological analysis

The tibiae that had the implants removed were fixed in 4% paraformaldehyde for 48 hours, washed in running water for 12 hours and placed in 7% EDTA solution for decalcification for a period 8 weeks with 3 changes of EDTA solution during the week at room temperature. Subsequently, the samples were washed and dehydrated in alcohol, diaphanized in xylol and embedded in paraffin. The sections were made parallel to the long axis of the site where the implants were placed. The 4-μm-thick slices were fixed in common (for haematoxylin-eosin staining) and silanized slides (for immunohistochemical analysis).

The histological description focused on the appearance of the bone tissue with emphasis on the bone remodelling and maturation process. The evaluations were performed by a trained and blinded rater (GJO) for the experimental groups using an optical microscope (DM 2500, Leica Reichert & Jung products, Wetzlar, Germany) with a magnification of 100X and 200X.

### Micro CT analysis

The right tibiae were scanned by a micro-CT scanner (Skyscan, Aatselaar, Belgium) with the following parameters: camera pixel: 12.45; X-ray tube power: 65 kVP, X-ray intensity: 385 μA, integration time: 300 ms, filter: Al-1 mm and voxel size: 18 μm. The images were reconstructed, spatially repositioned and analyzed by specific software (NRecon, Data Viewer, CTAnalyser, Aatselaar, Belgium). The region of interest (ROI) was defined as a 0.5-mm circular region around the entire diameter of the implant. This ROI was defined as the total volume (0.5 mm margin around implants - 4.5 mm x 3.2 mm). As the implants placed did not receive cover screws, in some cases, there was bone formation inside the prosthetic platform. A second ROI for the removal of the platform volume was defined in order to not interfere with the volume of mineralised tissue analysis in this osseous formation,. With the results obtained in the two ROIs, it was possible to define the volume of the mineralized tissues using the following equation: Total Volume − Platform Volume = Volume of mineralized tissues. The threshold used in the analysis was 25-90 shades of grey, and the values of the volume of mineralised tissues around the implants were obtained as a percentage.^25^ A trained rater blinded to the experimental groups performed this analysis (FEP).

### Histometry

The tibiae that underwent microtomographic analysis were used for histomorphometric analysis. The samples were dehydrated in a growing series of ethanol (60-100%) and infiltrated and polymerised in light-curing resin (Technovit 7200 VLC, Kultzer Heraus GmbH & CO, Wehrheim, Germany). The blocks containing the implant and the bone tissue were cut at a central point using a wear-and-tear system (Exakt Apparatebeau, Hamburg, Germany). The final sections were approximately 45 μm thick, stained with Stevenel blue associated with acid fuchsin and analyzed under an optical microscope (DIASTAR - Leica Reichert & Jung products, Wetzlar, Germany) at a magnification of 100X. Histomorphometric evaluations were performed with image analysis software (ImageJ, San Rafael, CA, USA). The percentages of bone-implant contact (% BIC) and bone area between the threads (% BBT) were separately evaluated at the first six threads of the implants manually without establishing thresholds in the software.^18^ We also performed an analysis of the percentage of bone and biomaterial in the region of the six threads near to the implants. These analyses were performed by a blinded and trained rater (FEP).

### Immunochemistry analysis

Immunohistochemistry evaluation was performed to identify and to localize the expression of bone remodelling-related proteins: osteocalcin (OCN), bone morphogenic protein 2 (BMP2), and alkaline phosphatase (ALP). The histological sections were mounted on silanized slides, followed by routine laboratory procedures for deparaffinization and rehydration. Subsequently, the sections were subjected to nonspecific epitope blockade with the application of hydrogen peroxide block for 10 minutes and protein block for 30 minutes (Spring Bioscience, Inc., Pleasanton, USA). Then, the sections were incubated for 16 hours in primary antibodies against OCN (1:400), BMP2 (1:400), and ALP (1:800) (Abcam, São Paulo, Brazil). As negative control, the histological sections were treated with 1% PBS. Subsequently, the sections were treated with the conjugate and HRP conjugate and stained with DAB (Spring Bioscience, Inc., Pleasanton, USA). The sections were counterstained with Carrazi haematoxylin solution for visualization of the cell nuclei. The images were obtained with a camera coupled to a light microscope (Leica-Reichert Diastar Products & Jung, Wetzlar, Germany) with a magnification of 200X. The analysis of the expression of proteins was performed in the area of the bone near to the first six threads of the implants with a protein-labeled extension index:^26^ (0) without labelling (0% of cells/matrix); (1) weak labeling (<25 % of cells/matrix); (2) moderate labelling (<50% of cells/matrix); (3) strong labeling (<75 % of cells/matrix). The analyses were performed by a blinded and trained rater (GJO).

### Statistical analysis

The data generated by the histometric, tomographic, and biomechanical analyses are numerical data; thus, they were subjected to the Shapiro-Wilk Normality test to evaluate the normal distribution according to the central distribution theorem. Data from biomechanical and immunohistochemical analysis were not normally distributed, thus the non-parametric Kruskal-Wallis test complemented by the Dunn test were used for the comparison between groups, and the Mann-Whitney test was used to evaluate the data within each group after varying the bone substitute and the experimental period. The other data were normally distributed, thus a parametric two-way ANOVA complemented by Tukey’s test were used to evaluate the data between the groups considering the relation between bone substitute and LLLT protocol used, whereas the independent t-test was used to compare data within each group after varying the experimental period. GraphPad Prism 6 software (San Diego, CA, USA) was used for the statistical tests. All statistical tests of this study were carried out with a 5% significance level.

## Results

All animals tolerated the surgical procedures and showed no suffering, weight loss or death during the experimental period. For the sample size estimation, the histological data of %BIC from a previous preclinical study evaluating the effect of LLLT applied to the grafted area of the osseointegration implant was used.^18^ The minimum difference between the averages of groups – where significant differences were found – was 12.85 %, with a standard deviation of 4.83 %. Therefore, the sample size of seven animals per group was sufficient for the application of the statistical tests with error type α of 0.05 and power 1-β greater than 0.90. The descriptive data of biomechanical and immunochemistry analyses are presented as mean [median] ± standard deviation, whereas the descriptive data of the micro-CT and histometric analyses are presented as mean ± standard deviation. The micro-CT, histometry, and immunohistochemistry analyses were repeated by the raters in 10 rats, and the data correlation was higher than 0.90.

### Biomechanical analysis

**Figure 2:**
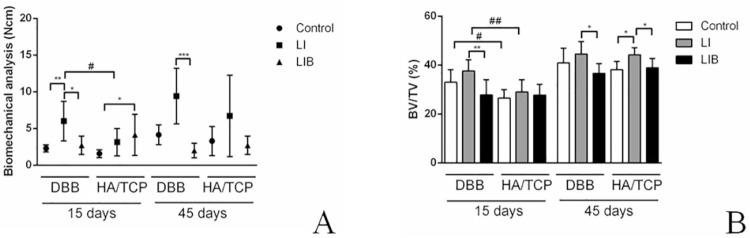
A) Representative graphs of the median and 25th and 75th percentiles of the biomechanical analysis. * p <0.05; ** p <0.01; ***p<0.001 – Significant differences between the different protocols of LLLT (Control, LI, and LIB) - Kruskal-Wallis test complemented by the Dunn test; #p <0.05 – Significant differences between the different bone substitutes - Mann-Whitney U-test. B) Representative graphs of the mean and standard deviation of the micro-CT analysis data. * p <0.05; ** p <0.01 - Significant differences between the different protocols of LLLT (Control, LI, and LIB); #p <0.05; ## p <0.01 – Significant differences between the different bone substitutes – Two-way ANOVA complemented by Tukey’s test

It was observed that the DBB-LI group (6.00 [5.00] ± 2.70 Ncm at 15 days and 9.42 [9.00] ± 3.78 Ncm at 45 days) presented higher removal torque values than those of the DBB group at 15 days (2.28 [2.00] ± 0.48 Ncm) and the DBB-LIB group (2.71 [2.00] ± 1.25 Ncm at 15 days and 2.00 [2.00] ± 1.00 at 45 days) at both periods of evaluation (p<0.05). The HA/TCP-LIB group had a higher removal torque (4.14 [3.00] ± 2.79 Ncm) compared with the HA/TCP group (1.57 [2.00] ± 0.53 Ncm) at 15 days (p<0.05). Furthermore, the DBB-LI group (6.00 [5.00] ± 2.70 Ncm) presented higher removal torque values than the HA/TCP-LI group (3.14 [2.00] ± 1.86 Ncm) at 15 days (p<0.05) (Figure 2A).

### Micro CT analysis

The volume of mineralized tissues around the implants increased in the 45-day period compared with the 15-day period in all groups (p<0.05). The DBB-LI group (37.56 ± 4.64 % at 15 days and 44.53±5.07 % at 45 days) presented higher volumes of mineralized tissues than those of the DBB-LIB group (27.77±6.27 % at 15 days and 36.63±3.92 % at 45 days) at both periods of evaluation (p<0.05), whereas the HA/TCP-LI group (44.16±2.93%) had a higher volume of mineralized tissues than the HA/TCP group (38.11±3.41%) and HA/TCP-LIB group (38.86±3.86%) at 45 days (p<0.05). Furthermore, the DBB (32.95±5.15 %) and DBB-LI groups (37.56±4.64 %) presented higher volumes of mineralized tissues than the HA/TCP (23.46±3.51 %) and HA/TCP-LI groups at 15 days (28.99±5.00%), respectively (Figure 2A).

### Descriptive histology and histometry

**Figure 3:**
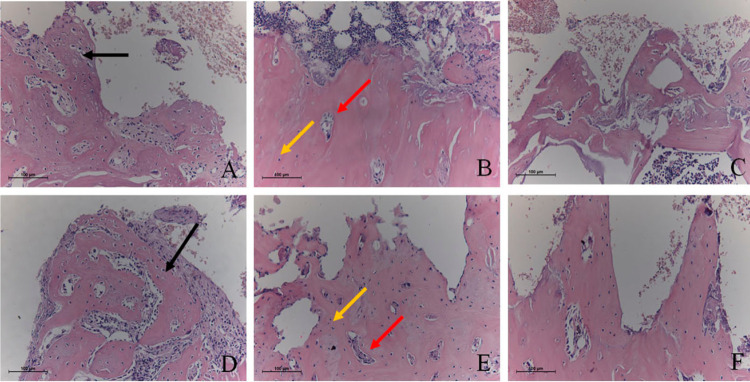
Representative images of decalcified histological sections. A) DBB and DBB-LI groups at 15 days; B) DBB-LIB group at 15 days; C) DBB groups at 45 days; D) HA/TCP and HA/TCP-LI group at 15 days; B) HA/TCP-LIB group at 15 days; C) HA/TCP groups at 45 days. (HE-100x magnification). At 15 days, it was verified that the new bone associated with the biomaterials in the DBB, HA/TCP, DBB-LI, and HA/TCP-LI groups presented an immature bone appearance associated with rounded osteocytes and active osteoblasts (black arrows), the formation of Haversian channels and a large number of new blood vessels (red arrows). The DBB-LIB and HA/TCP-LIB groups showed a more mature appearance, with flattened osteocytes and well-formed Haversian channels (yellow arrows). At 45 days, the presence of mature bone was observed in all groups, with Haversian channels in association with flattened osteocytes and well-established lamellar bone. In all groups and evaluation periods, the presence of biomaterials in contact with the neoformed bone or the connective tissue was observed

After 15 days, it was observed that the DBB, HA/TCP, DBB-LI, and HA/TCP-LI groups presented a large presence of immature bone associated with rounded osteocytes and active osteoblasts. Furthermore, the presence of Havers channels attested the formation of a large number of new blood vessels. The DBB-LIB and HA/TCP-LIB groups had bone tissue with more mature appearance, flattened osteocytes, well-formed Havers channels, and large presence of reversion lines and blood vessels. At 45 days, no differences were observed between the groups in relation to the histological aspect characterized by the presence of mature bone, with Havers channels associated with flattened osteocytes, and well-established lamellar bone. In all groups and evaluation periods, the presence of biomaterials in contact with the bone and connective tissue without the presence of significant inflammatory reactions were verified (Figure 3).

**Figure 4:**
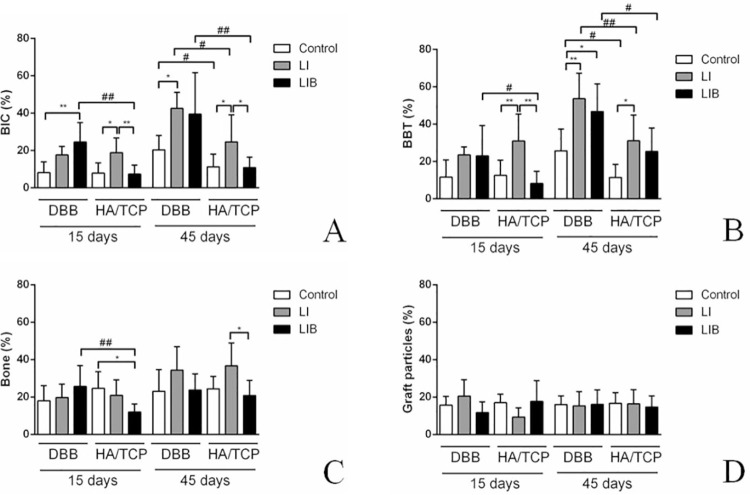
Representative graphs of the mean and standard deviation of the histometric analysis data. A) %BIC; B) %BBT; C) % bone; D) % biomaterial. * p<0.05; ** p<0.01 - Significant differences between the different protocols of LLLT (Control, LI, and LIB); # p <0.05; ## p<0.01 - Significant differences between the different bone substitutes – Two-way ANOVA complemented by Tukey’s test

Regarding the histometric analysis, a higher %BIC was observed in the DBB-LI group at 45 days (42.48±8.55%) and in the DBB-LIB group at 15 days (25.56±10.42%) compared with the DBB group (8.15±5.69% at 15 days and 20.32±7.69% at 45 days). The HA/TCP-LI group (18.79±7.90% at 15 days and 24.59±14.48% at 45 days) had a higher %BIC than the HA/TCP group (7.89±5.47 % at 15 days and 11.21±6.82 % at 45 days) and the HA/TCP-LIB group (7.27± .89% at 15 days and 10.86±5.50% at 45 days) at both periods of evaluation (p<0.05). Furthermore, the DBB-LIB group (25.56±10.42%) presented a higher %BIC than the HA/TCP-LIB group at 15 days (7.27±4.89%) (p<0.05), and the DBB (20.32±7.69%), DBB-LI (42.48 ± 8.55 %), and DBB-LIB (39.41±22.21%) groups presented a higher %BIC than the HA/TCP (11.21±6.82%), HA/TCP-LI (24.59±14.48%), and HA/TCP-LIB (10.86±5.50%) groups at 45 days (p<0.05), respectively (Figure 4A).

Regarding the %BBT, it was shown that the DBB-LI group (53.54±13.68%) and the DBB-LIB group (46.70±14.74%) had higher values for this parameter than the DBB group (25.64±11.70%) at 45 days. It was also shown that the HA/TCP-LI group (30.89±14.40% at 15 days and 31.00±13.75% at 45 days) had higher %BBT than the HA/TCP group (12.57±8.11% at 15 days and 11.37±7.09% at 45 days) at both experimental periods and a higher %BBT than the HA/TCP-LIB group (8.13±6.59%) at 15 days (p<0.05). Moreover, the DBB-LIB group (22.28±16.29%) presented higher %BBT than the HA/TCP-LIB at 15 days (8.13±6.59%) (p<0.05), and the DBB (25.64±11.70%), DBB-LI (53.54±13.68%), and DBB-LIB (46.70±14.74%) groups presented higher %BBT than the HA/TCP (11.37±7.09%), HA/TCP-LI (31.00±13.75%), and HA/TCP-LIB (25.33±12.56%) groups at 45 days (p<0.05), respectively (Figure 4B).

**Figure 5:**
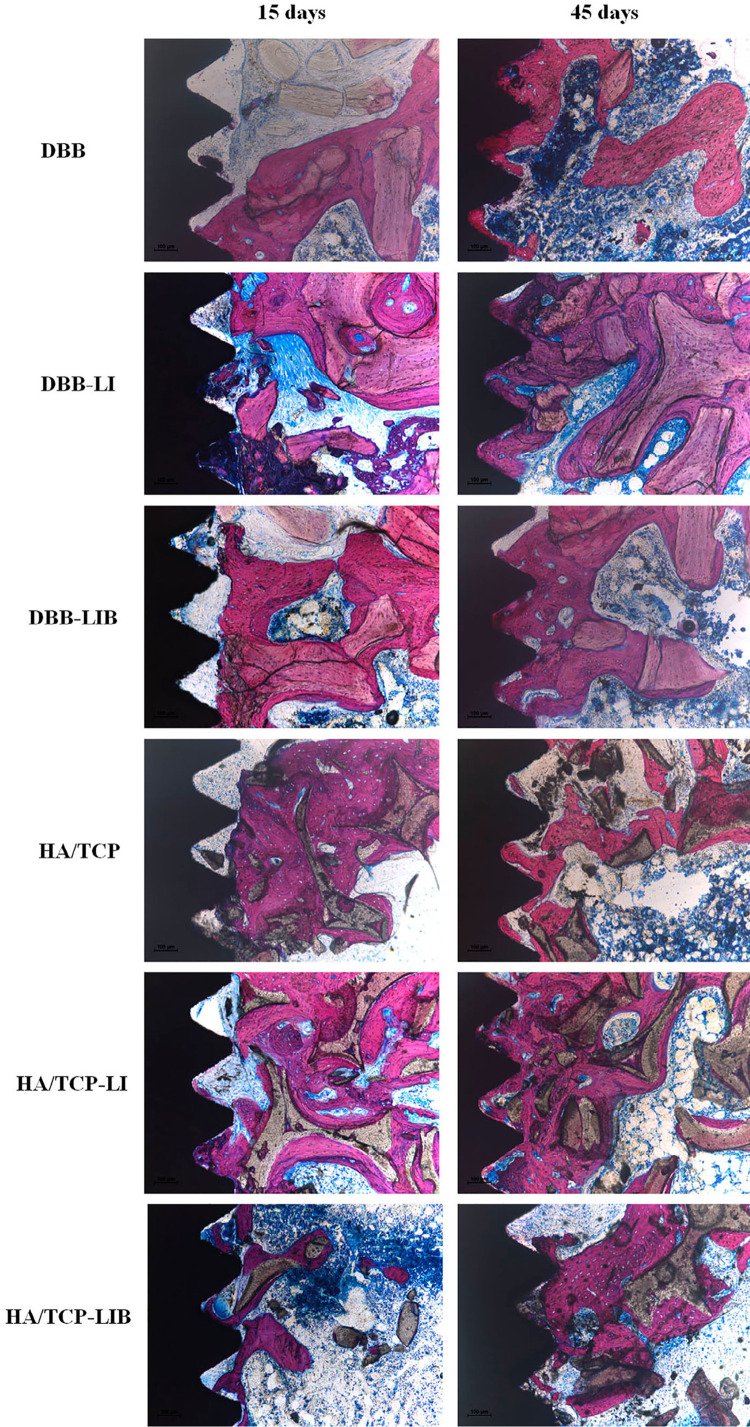
Representative images of non-decalcified histological sections of each group showing the presence of bone substitutes attached to the implant surfaces through a bridge of neoformed bone tissue (Stevenel’s blue and acid fuchsin-100x magnification)

In relation to the amount of bone and biomaterial in the grafted areas near the implants, a greater amount of bone was verified in the HA/TCP group at 15 days (24.67±8.87%) and in the HA/TCP-LI group at 45 days (36.67±12.26%) compared to that of the HA/TCP-LIB group (12.06±4.15% at 15 days and 20.85±8.05% at 45 days) (p<0.05). The DBB-LIB group (25.70±11.05%) presented more bone than the HA/TCP-LIB group (12.06±4.15%) at 15 days (p<0.05) (Figure 4C). The amount of biomaterial was not different between the groups evaluated (Figure 4D). Figure 5 shows representative images of the non-decalcified histological sections of all the groups.

### Immunochemistry analysis

**Figure 6:**
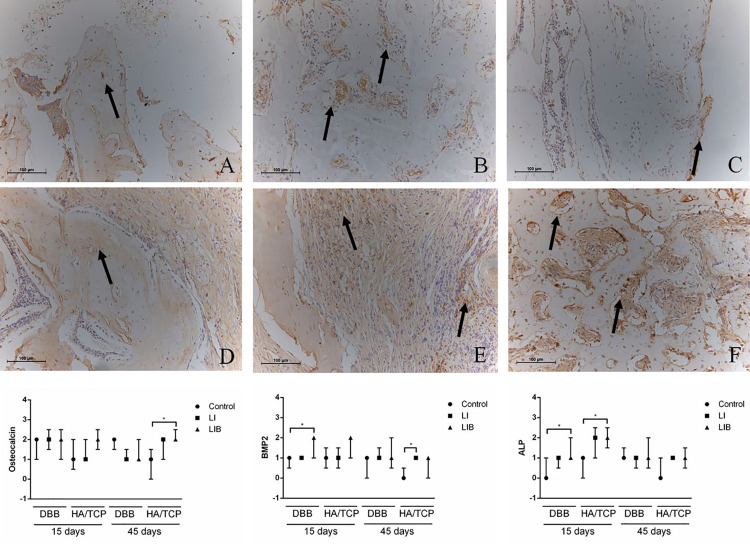
Representative images and graphs of the expression analysis of the OCN, BMP2, and ALP proteins. Protein expression in the non-irradiated groups (A-C). A) OCN at 45 days; B) BMP2 at 15 days; C) ALP at 15 days. Expression of the proteins in the irradiated groups (LI and LIB) (D-F). D) OCN at 45 days; E) BMP2 at 15 days; F) ALP at 15 days (200x magnification). * p<0.05 - Higher protein expression than the non-irradiated groups. Kruskal-Wallis test complemented by the Dunn test

The HA/TCP-LIB group (2.20 [2.00]±0.44) had higher expression of OCN than the HA/TCP group (0.80 [1.00]±0.83) at 45 days. Regarding the BMP2 expression, it was verified that the DBB-LIB group (1.60 [2.00]±0.54) expressed higher amounts of this protein than the DBB group (0.80 [1.00]±0.44) at 15 days. The HA/TCP-LI group (1.00 [1.00]±0.00) had higher BMP2 expression than the HA/TCP group (0.20 [0.00]±0.44) at 45 days. Furthermore, a greater expression of ALP was observed in the DBB-LIB (1.40 [1.00]±0.54) and HA/TCP-LIB groups (2.00 [2.00]±0.70) than in the DBB (0.40 [0.00]±0.54) and HA/TCP groups (0.60 [1.00]±0.54) at 15 days (Figure 6).

## Discussion

This study showed that LLLT improves osseointegration process in areas grafted with DBB and HA/TCP, but this effect was greater when the irradiation protocol was used only after implant placement, whereas the use of LLLT at two time points (after the graft procedures and after implant placement) demonstrated limited superiority in relation to non-irradiated groups. Notably, the histometric data presented significant differences that were slightly different than the biomechanical and micro-CT data since this model evaluated osseointegration in a 2D view, the biomechanical analysis evaluated osseointegration in an indirect way, and micro-CT was not able to evaluate the BIC because of the artefacts induced by the implants and bone substitutes.^18,25^

The use of LLLT after implant placement in areas grafted with DBB increased the removal torque and the volume of mineralized tissues around the implants compared to the double irradiation protocol (LIB). Moreover, there was an increased volume of mineralized tissues in implants placed in areas grafted with HA/TCP that were submitted to LLLT after implant placement compared with non-irradiated implants and the areas where the double irradiation protocol (LIB) was performed. These data demonstrate that the benefits of LLLT use are dose-dependent and that double irradiation reduces the beneficial effects of LLLT. This phenomenon was previously described in a study where increased irradiation dosage (16 J in two sessions) impaired the proliferation, migration, ATP activity, and viability of human skin fibroblast cells.^27^ Furthermore, Altan, et al.^3^ (2015) demonstrated that increased LLLT dosage (198 J in five sessions) reduced bone tissue formation in a model of hard palate expansion in rats. The dosage used in our (28 J in seven sessions in the LI group) was effective in improve bone repair in areas grafted with different osteoconductive biomaterials in different preclinical models^20,22^ as well as improving osseointegration in areas of native^20,28^ and grafted bone, and this may be the reason for the better outcomes of this protocol compared with the LIB protocol (56 J in 14 sessions) used in this study. However, previous studies demonstrated an improvement in the osseointegration of implants placed in grafted areas, where the double LLLT protocol was applied (4-184 J).^16,17^ Most likely, the different animal models of these preclinical studies (rabbit tibiae^17^ and maxillary sinus of sheep^16^) may explain these contradictory outcomes since in the tibia of rats, the distance required for the laser energy induce some effects on the bone defects is smaller than in the models mentioned above, and probably the energy required to reach the bone defect could be higher in the model used in this study. Considering that the supposedly beneficial LLLT dosage for bone repair associated with the treatment of bone defects using biomaterials, positively influencing the osseointegration of implants is unknown, demonstrates that this topic requires more research.

The histological analysis of the decalcified sections showed that the group in which the double irradiation was performed presented a more evident pattern of bone maturation in the 15-day period than the other groups, and this fact may have occurred because of the LLLT treatment in the grafted area, which promoted an acceleration of bone maturation in the grafted area compared with the other groups, a fact that corroborates the findings of other preclinical studies.^19,20,22,29^ A preclinical study that evaluated the effect of LLLT on a model of Teflon domes filled with HA/TCP and DBB that were fixed in the lateral surface of the mandibular ramus of rats demonstrated that the use of LLLT in grafted areas increases the bone formation,^22^ and this finding may be the explanation for the greater bone maturation found after LLLT on the grafted areas shown in our study.

Another significant finding of this study was that LLLT increased the osseointegration of implants in grafted areas and that this effect was higher, especially when LLLT was used only after implant installation. It has been previously shown that LLLT increases osseointegration of implants placed in native bone in healthy^13,14^ and osteopenic animals,^28^ facilitating the osseointegration of implants placed in the maxillary sinus of sheep grafted with autogenous bone grafts^16^ and improving the osseointegration of implants placed in the tibiae of rabbits grafted with blocks of deproteinized bovine bone.^17^ The preclinical study by Oliveira, et al.^18^ (2020) – that evaluated the effect of LLLT applied on noncritical defects in the tibia of rats grafted with HA/TCP and DBB prior to implant placement – promoted an increase in the %BIC and %BBT. To our knowledge, this is the first study showing that LLLT improves the osseointegration of implants placed in grafted areas when irradiation was performed only after implant placement.

The effects of LLLT were also dependent on the type of bone substitute used for the grafting procedures, and the implant placements in the defects grafted with DBB presented a better pattern of osseointegration than the implant placements in HA/TCP grafted areas. Both bone substitutes tested in this study have shown good clinical outcomes,^30^ and some histological studies show that areas grafted with DBB and HA/TCP present no differences regarding bone formation.^31-33^ However, a clinical study showed that biopsies harvested from the maxillary sinus grafted with DBB presented higher levels of osteoconduction than biopsies from the maxillary sinus grafted with HA/TCP.^32^ It is likely that this better pattern of osteoconduction justifies the better pattern of osseointegration obtained in areas grafted with DBB that were treated with LLLT.

The use of LLLT is associated with proliferative tissue effects in connective tissue,^34^ increased angiogenesis,^35^ and an improvement in osteoblastic differentiation and activity.^8,36,37^ Indeed, an increase in the expression of OCN, BMP2, and ALP, significant mediators in the formation and maturation of bone tissue, were observed in this study.^38-40^ Previous studies have demonstrated that LLLT irradiation at infrared wavelength increased the expression of ALP, OCN, BMP2, and Jagged 1 in HA/TCP- and DBB-grafted areas.^22^ Furthermore, Kim, et al.^41^ (2009) showed that LLLT increased the expression of RANK, RANKL, and OPG in critical-sized calvarial defects of rats grafted with DBB, and this finding was related to the increase in the stimulus of bone remodelling. These events may be associated with the increase in osseointegration observed in our study because of the LLLT use.

The results presented in this study raise the possibility of using LLLT in areas with poor bone quality as a way to improve osseointegration. It is necessary to compare the effects of the infrared laser with the red laser to evaluate whether there are differences in the use of these two distinct wavelengths since the results presented in the literature to this date are conflicting.^16,29,42,43^ Clinical studies evaluating LLLT with infrared lasers on osseointegration are also required. Only one clinical study evaluated the effect of infrared laser LLLT on osseointegration, and no differences were found in achieving the secondary stability of implants. In the initial period of evaluation, the stability obtained by the implants installed in the control and laser groups reached high values of stability.^23^ However, in this study the implants were installed in the posterior region of the mandible, and this area is not considered an area with poor bone quality.

This study presents some drawbacks that need to be considered in the our data interpretation. The type of defect tested in this study was a noncritical size defect because of the limitations of space to perform a critical-sized defect in the tibia that enables implant placement, so the type of defect tested may be less challenging than the conditions normally present in humans. The absence of a control group in which the implants should be placed in native bone limits the comparison of our data with the ideal conditions for implant placement, since this condition may be a more conventional positive control. Furthermore, it is necessary to compare the LLLT performed only after the grafting procedures to understand if the LLLT performed at two different times (after the grafting procedure and implant placement) presents inferior outcomes compared with the LLLT applied just after the implant placement, and also to assess whether these outcomes were due to the high doses of irradiation or to previous impact of the LLLT in the grafted area, and this evaluation was not possible in this study. Finally, the method used in this study to evaluate protein expression (immunohistochemistry) is more susceptible to systematic errors than other techniques used to evaluate protein expression (e.g., PCR, Western blotting), and the evaluation of the mechanisms of LLLT on bone formation in grafted areas should be performed with these techniques in the future.

## Conclusion

Thus, it can be concluded that LLLT performed after implant placement in the grafted areas enhances osseointegration. However, the LLLT irradiation protocol after the grafting procedures associated with LLLT after implant placement showed limited improvement in osseointegration compared with the non-irradiated groups.
